# Molecular subtypes of epilepsy associated with post-surgical seizure recurrence

**DOI:** 10.1093/braincomms/fcad251

**Published:** 2023-09-30

**Authors:** Courtney E Hershberger, Shreya Louis, Robyn M Busch, Deborah Vegh, Imad Najm, Peter Bazeley, Charis Eng, Lara Jehi, Daniel M Rotroff

**Affiliations:** Department of Quantitative Health Sciences, Lerner Research Institute, Cleveland Clinic, Cleveland, OH 44195, USA; Cleveland Clinic Lerner College of Medicine, Cleveland Clinic, Cleveland, OH 44195, USA; Cleveland Clinic Lerner College of Medicine, Cleveland Clinic, Cleveland, OH 44195, USA; Epilepsy Center, Neurological Institute, Cleveland Clinic, Cleveland, OH 44195, USA; Genomic Medicine Institute, Lerner Research Institute, Cleveland Clinic, Cleveland, OH 44195, USA; Epilepsy Center, Neurological Institute, Cleveland Clinic, Cleveland, OH 44195, USA; Cleveland Clinic Lerner College of Medicine, Cleveland Clinic, Cleveland, OH 44195, USA; Epilepsy Center, Neurological Institute, Cleveland Clinic, Cleveland, OH 44195, USA; Department of Quantitative Health Sciences, Lerner Research Institute, Cleveland Clinic, Cleveland, OH 44195, USA; Cleveland Clinic Lerner College of Medicine, Cleveland Clinic, Cleveland, OH 44195, USA; Genomic Medicine Institute, Lerner Research Institute, Cleveland Clinic, Cleveland, OH 44195, USA; Center for Personalized Genetic Healthcare, Community Care and Population Health, Cleveland Clinic, Cleveland, OH 44195, USA; Department of Genetics and Genome Sciences, Case Western Reserve University School of Medicine, Cleveland, OH 44195, USA; Cleveland Clinic Lerner College of Medicine, Cleveland Clinic, Cleveland, OH 44195, USA; Epilepsy Center, Neurological Institute, Cleveland Clinic, Cleveland, OH 44195, USA; Department of Quantitative Health Sciences, Lerner Research Institute, Cleveland Clinic, Cleveland, OH 44195, USA; Cleveland Clinic Lerner College of Medicine, Cleveland Clinic, Cleveland, OH 44195, USA; Endocrinology and Metabolism Institute, Cleveland Clinic, Cleveland, OH 44195, USA; Center for Quantitative Metabolic Research, Cleveland Clinic, Cleveland, OH 44195, USA

**Keywords:** epilepsy surgery, resection, RNA, seizure freedom, clustering

## Abstract

Approximately 50% of individuals who undergo resective epilepsy surgery experience seizure recurrence. The heterogenous post-operative outcomes are not fully explained by clinical, imaging and electrophysiological variables. We hypothesized that molecular features may be useful in understanding surgical response, and that individuals with epilepsy can be classified into molecular subtypes that are associated with seizure freedom or recurrence after surgical resection. Pre-operative blood samples, brain tissue and post-operative seizure outcomes were collected from a cohort of 40 individuals with temporal lobe epilepsy, 23 of whom experienced post-operative seizure recurrence. Messenger RNA and microRNA extracted from the blood and tissue samples were sequenced. The messenger RNA and microRNA expression levels from the blood and brain were each subjected to a novel clustering approach combined with multiple logistic regression to separate individuals into genetic clusters that identify novel subtypes associated with post-operative seizure outcomes. We then compared the microRNAs and messenger RNAs from patient blood and brain tissue that were significantly associated with each subtype to identify signatures that are similarly over- or under-represented for an outcome and more likely to represent endophenotypes with common molecular aetiology. These target microRNAs and messenger RNAs were further characterized by pathway analysis to assess their functional role in epilepsy. Using blood-derived microRNA and messenger RNA expression levels, we identified two subtypes of epilepsy that were significantly associated with seizure recurrence (clusters A1 and B4) (adjusted *P* < 0.20). A total of 551 microRNAs and 2486 messenger RNAs were associated with clusters A1 and B4, respectively (adjusted *P* < 0.05). Clustering of brain–tissue messenger RNA expression levels revealed an additional subtype (C2) associated with seizure recurrence that had high overlap of dysregulated messenger RNA transcripts with cluster B4. Clusters A1, B4 and C2 also shared significant overlap of subjects, which altogether suggests a coordinated mechanism by which microRNA and messenger RNA transcripts may be related to seizure recurrence. Epileptic subtypes A1, B4 and C2 reveal both known and novel microRNA and messenger RNA targets in seizure recurrence. Furthermore, targets identified in A1 and B4 are quantifiable in pre-operative blood samples and could potentially serve as biomarkers for surgical resection outcomes.

## Teaser

Hershberger *et al*. apply a novel clustering approach to microRNA (miRNA) and messenger RNA (mRNA) expression data to identify two overlapping subtypes of patients with epilepsy that are significantly associated with post-operative seizure recurrence. Concurrent dysregulation of miRNAs and target genes between the two subtypes suggests a common mechanism associated with seizure recurrence.

## Graphical Abstract

**Graphical Abstract:**
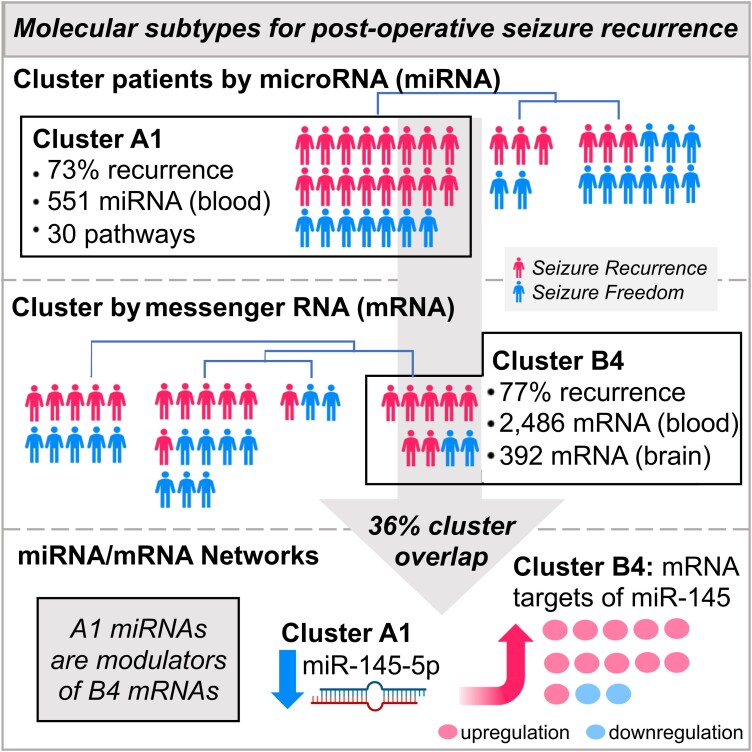

## Introduction

Epilepsy has a prevalence of 7.60 per 1000 people worldwide.^1^ For over 20 years, surgical resection for the treatment of temporal lobe epilepsy (TLE) has been recognized as superior to medical therapy^2^ and will be curative for 40–80% of candidates.^3–5^ However, surgery is underutilized and often recommended only after the failure of multiple anti-seizure medications (ASMs),^2^ despite the fact that duration of epilepsy is an independent predictor of post-operative seizure freedom.^6^ Evaluation of surgical candidates is complex, involving analysis of clinical and imaging data by a team of radiologists, neurologists and neurosurgeons.^7^ Models to predict surgical outcomes on clinical and imaging data such as sex, seizure frequency, type of surgery, pathological cause, MRI epilepsy duration and quantitative MRI volumetrics have ∼73% discrimination.^8–10^

Incorporation of molecular variables into clinical decision-making may further increase the discriminatory capabilities of existing prediction models for surgical outcomes. Recently, the value of genetic testing in conjunction with clinical variables for surgical candidacy has proven advantageous, and genomic single nucleotide polymorphisms have shown associations with surgical outcomes.^11,12^

In addition to DNA variants, mRNA transcripts and miRNAs have been assessed for association with post-surgical seizure freedom.^12,13^ When comparing brain transcriptomes from two individuals with late recurring seizures to transcriptomes from four individuals who remained seizure free post-resection, 29 target transcripts were identified.^13^ A second study, analysing mRNA and miRNA from brain tissue of 132 and 135 individuals, respectively, did not identify any individual miRNAs or mRNAs associated with surgical outcomes; however, several signalling and metabolic pathways associated with seizure recurrence were significantly enriched.^12^ This suggests that a collection of smaller and coordinated effects on the transcriptome could contribute to surgical outcome.

The levels of some transcripts can be correlated between brain tissue and blood samples, and recent studies have shown striking parallels between brain and blood transcriptomes that are associated with neuropathy and clinical deterioration.^14,15^ While miRNA panels have been evaluated as a diagnostic tool for epilepsy and drug-resistant epilepsy, to our knowledge, no studies have classified the circulating mRNA transcripts and miRNAs that differ between individuals with epilepsy who are rendered seizure free following surgery and those who experience post-operative seizure recurrence.^16^

In this paper, we explore the hypothesis that molecular features ascertained from RNA and miRNA expression in the blood and brain may be useful in understanding surgical response, and that individuals with epilepsy can be classified into molecular subtypes that are associated with seizure freedom or recurrence after surgical resection.

## Materials and methods

### Patient cohort and clinical data

**Figure 1:**
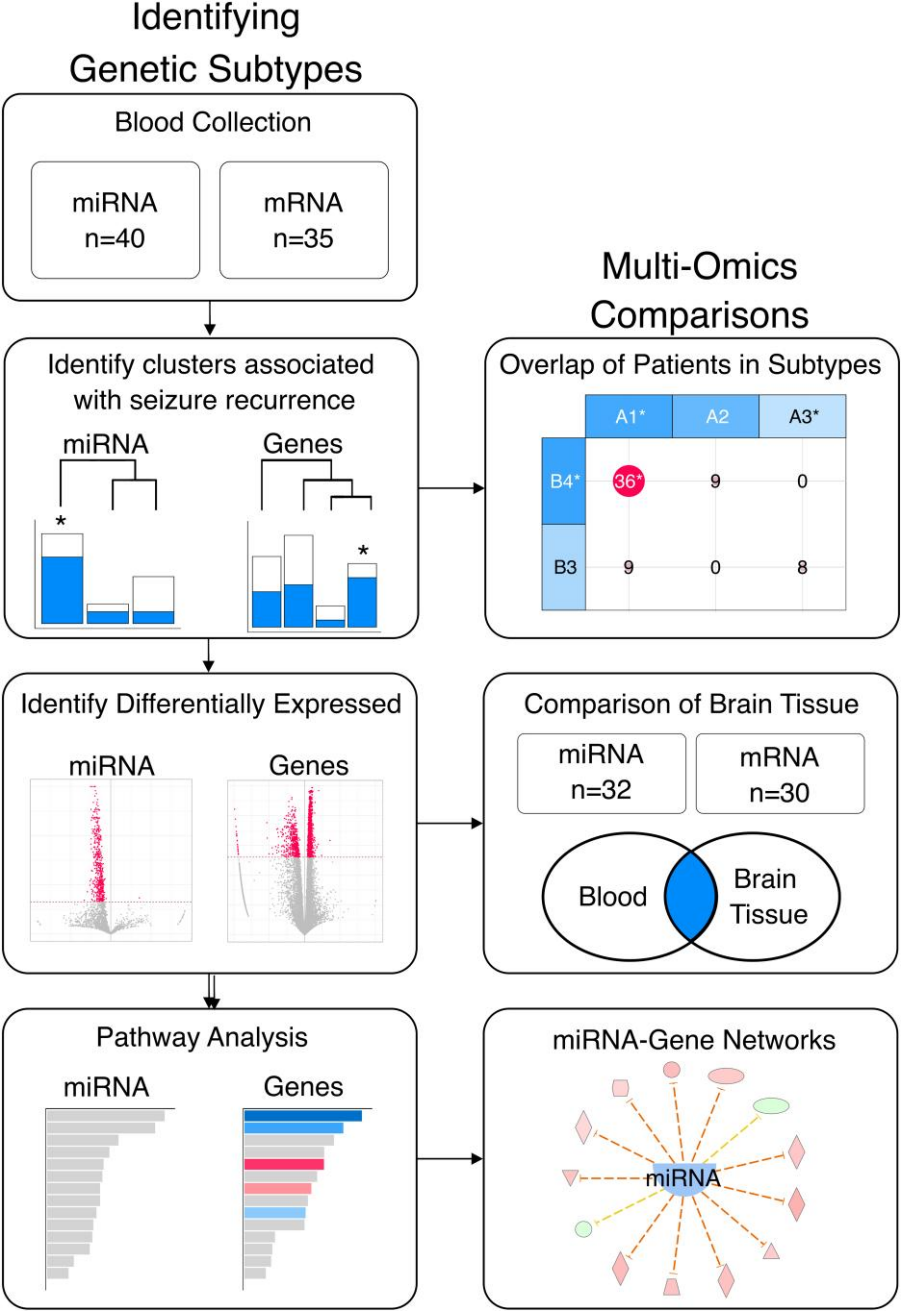
Study workflow depicting sample collection and data analysis.

Seizure freedom was defined by an Engel score of Ia or Ib recorded at least 1 year (11.6–281.3 months; median = 52.1; mean = 61.8) following surgical resection and was considered the primary outcome (Supplementary Fig. 1A). Blood samples were collected from 40 individuals: miRNA was sequenced from all 40 (including 17 who were seizure free for at least 1 year and 23 who experienced seizure recurrence), and mRNA was sequenced from 35 samples (16 seizure free and 19 with seizure recurrence) (Fig. 1). Brain tissue was also collected from 32 individuals out of the original cohort of 40 individuals. miRNA and mRNA were sequenced from all 32 (15 individuals were seizure free and 17 experienced seizure recurrence). All four data types were collected from 30 individuals (Supplementary Fig. 1B). Additional data, including patient-reported sex, pathology (i.e. hippocampal sclerosis, malformations of cortical development, no lesion and other), surgical side (i.e. dominant and nondominant) and epilepsy duration, were collected (Table 1). A detailed review of the study participants’ ASMs revealed that, overall, there were minimal ASM changes between the point of blood draw and surgery, suggesting that ASM changes were unlikely to contribute to our findings (Supplementary Table 1). Specifically, the ASM regimens remained unchanged during the interval between blood draw and brain surgery in 23/40 individuals. In the remaining 16/17, changes were limited to adding or removing a single ASM (details in Supplementary Table 2). All study participants provided informed consent, and the study was approved by the Cleveland Clinic Institutional Review Board (IRB 16-1539).

**Table 1:** Cohort characteristics of miRNA and mRNA blood-derived samples

miRNA blood	Seizure free (*n* = 17)	Seizure recurrence (*n* = 23)	Total (*n* = 40)
Overall cohort			
Age at surgery (years), mean (SD)	44.1 (13.4)	42.4 (12.5)	43.1 (12.7)
Epilepsy duration (years), mean (SD)	22 (10.8)	20.8 (15.4)	21.3 (13.5)
Female sex (%)	9 (52.9%)	10 (43.5%)	19 (47.5%)
White race (%)	17 (100%)	22 (95.7%)	39 (97.5%)
Dominant-sided surgery (%)	9 (52.9%)	12 (52.2%)	21 (52.5%)
Pathology (%)			
Hippocampal sclerosis	9 (52.9%)	13 (56.5%)	22 (55%)
Malformations of cortical development	1 (5.9%)	3 (13%)	4 (10%)
Other	4 (23.5%)	2 (8.7%)	6 (15%)
No lesion	3 (17.6%)	5 (21.7%)	8 (20%)

mRNA blood	Seizure free (*n* = 16)	Seizure recurrence (*n* = 19)	Total (*n* = 35)
Overall cohort			
Age at surgery (years), mean (SD)	44.4 (13.8)	42.6 (13.5)	43.4 (13.4)
Epilepsy duration (years), mean (SD)	21.7 (11.1)	21.1 (16.7)	21.4 (14.1)
Female sex (%)	9 (56.2%)	7 (36.8%)	16 (45.7%)
White race (%)	16 (100%)	18 (94.7%)	34 (97.1%)
Dominant-sided surgery (%)	8 (50%)	9 (47.4%)	17 (48.6%)
Pathology (%)			
Hippocampal sclerosis	8 (50%)	10 (52.6%)	18 (51.4%)
Malformations of cortical development	1 (6.2%)	3 (15.8%)	4 (11.4%)
Other	4 (25%)	2 (10.5%)	6 (17.1%)
No lesion	3 (18.8%)	4 (21.1%)	7 (20%)

miRNA and mRNA tissue	Seizure free (*n* = 15)	Seizure recurrence (*n* = 17)	Total (*n* = 32)
Overall cohort			
Age at surgery (years), mean (SD)	43.8 (10.5)	42 (13.1)	42.9 (11.8)
Epilepsy duration (years), mean (SD)	23.1 (10.3)	23 (17.4)	23.1 (14.2)
Female sex (%)	8 (53.3%)	8 (47.1%)	16 (50%)
White race (%)	15 (100%)	16 (94.1%)	31 (96.9%)
Dominant-sided surgery (%)	7 (46.7%)	6 (35.3%)	13 (40.6%)
Pathology (%)			
Hippocampal sclerosis	9 (60%)	10 (58.8%)	19 (59.4%)
Malformations of cortical development	1 (6.7%)	1 (5.9%)	2 (6.2%)
Other	2 (13.3%)	2 (11.8%)	4 (12.5%)
No lesion	3 (20%)	4 (23.5%)	7 (21.9%)

### Sample collection and processing

Whole blood was collected from patients with TLE prior to surgical resection (median = 97 days) (Supplementary Fig. 1). Total RNA (+miRNA) was extracted from plasma samples using the standard TRIzol extraction protocol. Cells were lysed in TRIzol, and chloroform was added to the sample. Samples were centrifuged, and the upper aqueous phase was removed. Ice-cold isopropanol was added to precipitate the RNA, and the resulting pellet was washed twice in ice-cold 75% ethanol. The RNA was dissolved in RNAse-free water and quantitated via NanoDrop and Qubit assay, and DNAse I treatment was performed.

Tissue samples were collected during surgical resection and subsequently flash frozen as previously described.^12^ Total RNA (+miRNA) were extracted using a modified version of the miRNeasy Mini Kit from Qiagen. Tissues were homogenized in QIAzol Lysis Reagent using 5 mm stainless steel beads for 2 min at 20 Hz with a TissueLyser II (Qiagen). Chloroform was added to the homogenate, the upper aqueous phase removed, and 100% ethanol was added prior to transferring the mixture on to an RNeasy Mini column. RNA cleanup and DNase treatment were performed using reagents from the kit directly on the RNeasy Mini column. Quantification of RNA was performed via NanoDrop and Qubit assay.

### mRNA and miRNA sequencing

The TruSeq Stranded Total RNA Kit (Illumina, San Diego, CA) was used to deplete rRNA and prepare libraries of total mRNA for sequencing for blood and tissue samples. mRNA sequencing was performed by the Genomics Core of the Cleveland Clinic (Cleveland, OH) on an Illumina HiSeq 2000 (100 bp, paired-end). Raw sequences were assessed for quality, trimmed for adapters and filtered using Fastp, then aligned to the hg38, and quantified by Salmon v0.14.1.^17–19^ Raw counts were converted to transcripts per million using tximport and then collapsed by Hugo gene name, with sum of the counts representing all isoforms of each gene.^20^

The NEXTFLEX Small RNA-Seq Kit V3 (Perkin Elmer, Waltham, MA) was used to prepare libraries for miRNA sequencing from total RNA extracted from blood and tissue. The libraries were sequenced by the Genomics Core of the Cleveland Clinic (Cleveland, OH, USA). Quality was assessed using FastQC, and raw reads were processed using the nf-core smrnaseq pipeline.^21,22^ miRNAs were aligned to mature and stem–loop miRNA sequences from miRbase v22.1 using Bowtie2 v1.2.2.^23,24^ The aligned miRNA sequences were then quantified using idxstats from Samtools and converted to counts per million (CPM) using edgeR.^25^

### Data processing and quality control

All data analysis was performed using the statistical software R-4.0.0.^26^ Expression data represented as counts for miRNA and transcripts per million for mRNA were scaled and centred within each respective processing and/or sequencing batch to correct for potential batch effects. miRNA and mRNAs with low variance, defined as ≥19:1 ratio of the most common value to the second most common value and ≤10% of the within-sample values, were excluded from subsequent analyses using the ‘caret’ package.^27^ After removal of low-variance miRNAs and mRNAs, the final matrices were scaled and centred.

### Statistical analyses

#### Patient subtyping

##### Overview

Patient subtypes were identified by performing unsupervised hierarchical clustering of all quantified blood miRNAs (2016) and mRNAs (16 683) and brain miRNAs (2247) and mRNAs (17 363) that passed quality control. The clustering of the individuals using all miRNAs and mRNA transcripts was performed without knowledge of seizure recurrence status and produced a single dendrogram, describing the similarity of the molecular profiles of the patients. The resulting dendrogram was then iteratively divided into clusters; the first split formed two clusters, the next split formed three clusters and so forth, until a maximum of 10 clusters were formed. For each split of the dendrogram, the clusters were examined to determine which cluster best separated patients who were seizure free from those who were not (i.e. clusters in which there was an over- or under-representation of individuals whose seizures recurred post-surgery) (Supplementary Fig. 2).

##### Clustering parameter optimization

Hierarchical clustering of all miRNA and mRNA transcripts was performed 12 times, for each combination of distance measures (i.e. Manhattan, Canberra, binary, Minkowski and Euclidean) and similarity measures (i.e. Ward D, Ward D2 and Complete). The resulting dendrograms were split in a stepwise manner to identify the optimal number of clusters, based on the following steps:

- ‘Identification of significant clusters’: For each of the 12 dendrograms produced by the different similarity and distance metrics, the dendrogram was first partitioned into *n* = 2 clusters. Each cluster was then compared to all other clusters and tested for associations with seizure recurrence using a multiple logistic regression model. Models were adjusted for sex, pathology, surgical side and epilepsy duration as described in the ‘Patient cohort and clinical data’ section. The *P*-values were corrected for multiple hypothesis testing using a false discovery rate (FDR) approach. FDR < 0.2 was selected as an exploratory threshold for significance.^28^ If no association was found, the dendrogram was split again, and the process was repeated until a significant cluster was identified or a maximum of 10 clusters was reached.
- ‘Selection of the optimal number of clusters’: Once a significant cluster was identified among *n* clusters, the dendrogram was split again (*n +* 1), and each cluster was compared to the rest of the cohort and tested for an association with seizure recurrence using multiple logistic regression, as described above. If the minimum nominal *P*-value of *n* + 1 cluster associations was less than the nominal *P*-value of the *n* cluster associations, then the *n* + 1 number of clusters was defined as the optimal number of clusters. This process was repeated until the *n* + 1 cluster had a less significant *P*-value with *n* clusters. In this circumstance, the *n* number of clusters was considered to be optimal based on a reduction in statistical significance with further partitioning of the cohort (Supplementary Fig. 2).
- ‘Selection of the optimal clustering approach’: Once an optimal number of clusters was identified for each of the 12 combinations of similarity and distance measures, the clustering strategy that yielded the cluster with the lowest FDR was identified as the optimal subtype and was used in subsequent analysis.

#### Bootstrapping proportions of seizure recurrence in clusters

We estimated the confidence intervals (CI) around the proportions of seizure recurrences in the newly identified subtypes using a bootstrapping approach. Within each cluster associated with seizure outcomes, we performed 1000 bootstrapped samples, and the 95% confidence intervals for the proportion of individuals with seizure recurrence were derived. The bootstrapped distributions were also used to perform a Student’s *t*-test to assess whether the differences in seizure outcomes between the cluster of interest and the rest of the cohort were significant.

#### Overlapping clusters

Once subtypes were identified using the miRNA and mRNA data, as described above, clusters that were significantly over- or under-represented for seizure recurrence among each of the modalities were compared. A Fisher’s exact test for over-representation was performed to evaluate whether two clusters contained a statistically significant overlap of individuals, and cluster pairs with effect sizes in the same direction and *P* < 0.05 were considered to have significant overlap.

#### Blood and brain tissue miRNAs and mRNAs associated with significant clusters (clusters A1 and B4)

A Student’s *t*-test was used to identify molecular features that were significantly associated with seizure recurrence between the cluster of interest (A1 or B4) compared to the rest of the cohort (A2 + A3 and B1 + B2 + B3). The Benjamini–Hochberg method was used to correct for multiple hypothesis testing, and mRNAs or miRNAs with an FDR < 0.05 were considered statistically significant.^29^ To calculate the fold change (FC) of scaled and centred data, each mRNA and miRNA was linearly transformed by adding a minimum expression value and 0.001. Comparisons between statistically significant blood-derived and tissue-derived mRNA and miRNAs were tested for significant overlap using Fisher’s exact tests.

#### Pathway and Gene Set Enrichment Analysis

The miRNAs and mRNAs from the brain tissue of individuals in clusters A1 and B4 were assessed using Gene Set Enrichment Analysis. Significant mRNAs and miRNAs from blood samples were used to create custom pathways: A1_miRNA_DOWN, B4_mRNA_UP and B4_mRNA_DOWN. The differential expression (DE) analysis results comparing brain tissue miRNA in A1 compared to A2–A3 were ranked by FC. A1_miRNA_DOWN was tested for enrichment at the top or bottom of the ranked list. Similarly, the DE analysis results comparing brain tissue mRNA from cluster B4 to clusters B1–B3 were ranked by FC. B4_mRNA_UP and B4_mRNA_DOWN were tested for enrichment at the top or bottom of the ranked brain tissue gene list using the ‘fgsea’ R package.^30^

#### Pathway and upstream regulator analysis

Pathway analysis was performed on miRNAs and mRNAs that were differentially expressed in the blood of individuals in clusters A1 and B4 (FDR *P* < 0.05), respectively, using Ingenuity Pathway Analysis (IPA).^31^ The Upstream Regulator Analysis tool was used to approximate causal relationships between miRNAs and quantified mRNAs using mRNA B4 versus B1–B3 FC and *P*-values. The Upstream Regulator Analysis tests for over-representation using a Fisher’s exact test in molecular networks from the literature informed Ingenuity Knowledge Base. Next, the *Z*-score is calculated to describe the predicted direction of change. If the expression of the mRNA targets is consistent with the Knowledge Base, IPA predicts that the miRNA is more active in B4. If there is an anti-correlation with the Knowledge Base, IPA predicts that the miRNA is less active.^31^ We then compared these predicted miRNA activation scores based on mRNA expression in B4 versus B1–B3 (*P* < 0.05) with the miRNA expression levels in A1 versus A2-–A3 (Student’s *t*-test).

## Results

### miRNA identification of TLE patient subtypes with modified risk of post-surgical seizure outcomes

**Figure 2:**
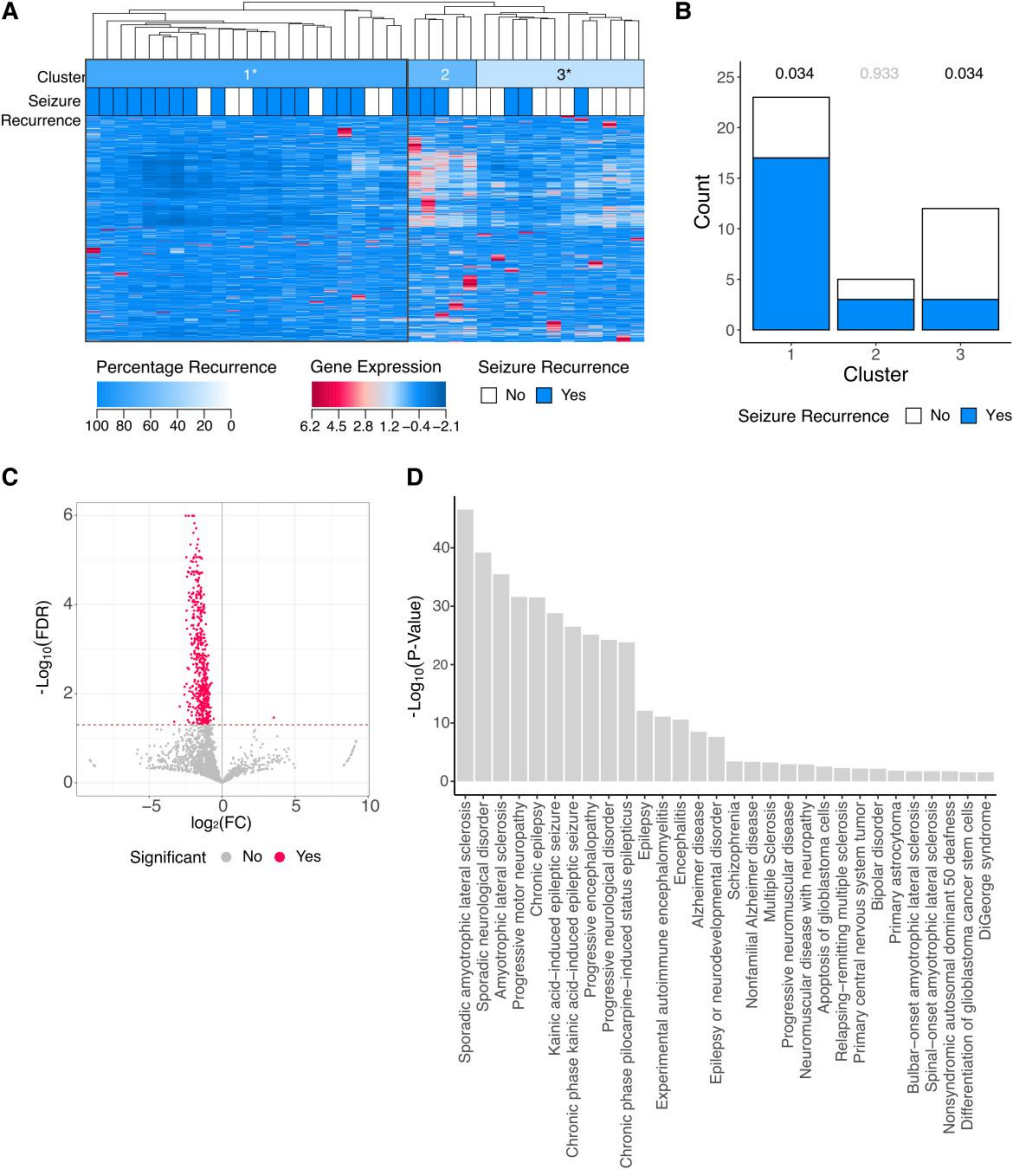
Identification of molecular subtype of epilepsy (A1), associated with seizure recurrence using miRNA-Seq data. (**A**) Semi-supervised hierarchical clustering of miRNA quantifications identifies cluster of individuals with a higher rate of seizure recurrence after surgical resection (cluster A1, multiple logistic regression, FDR *P* = 0.034). (**B**) The number of individuals in each cluster who experienced seizure freedom or seizure recurrence after surgical resection (multiple logistic regression, cluster A1 FDR *P* = 0.034, cluster A3 FDR *P* = 0.034). (**C**) Volcano plot depicting FC and FDR of 2016 miRNAs compared between cluster A1 and clusters A2–B3 (Student’s *t*-test, dotted line indicates FDR *P* = 0.05). (**D**) The neurological disease pathways (defined by IPA) that are enriched for the miRNAs which are significantly associated with cluster A1 (right-tailed Fisher’s exact test, *P* < 0.05).

The subtyping method was applied to miRNA and mRNA sequenced from patient blood samples. Hierarchical clustering of miRNAs with Canberra distance and complete similarity identified clusters most significantly associated with seizure outcome, and three clusters (A1–A3) were determined to be optimal (FDR < 0.05) (Fig. 2A; Supplementary Fig. 3A and B). Cluster A1 was associated with seizure recurrence when compared to clusters A2 + A3, 17/23 individuals in cluster A1 had post-operative seizures [74%, 95% CI (57–91%), FDR *P* = 0.03]. Cluster A3 was associated with seizure freedom when compared with clusters A1 + A2; 3/12 individuals in cluster A3 had post-operative seizures [25%, 95% CI (8–50%), FDR *P* = 0.03] (Fig. 2A and B; Supplementary Table 3). A total of 551 miRNAs were significantly different between cluster A1 and the rest of the cohort (clusters A2 + A3) (Fig. 2C; Supplementary Table 4) (FDR *P* < 0.05). These miRNAs were enriched for 30 neurological disease pathways including several pathways specific to epilepsy (Fig. 2D) (*P* < 0.05).

### mRNA identification of patient subtypes with modified risk of post-surgical seizure outcomes

**Figure 3:**
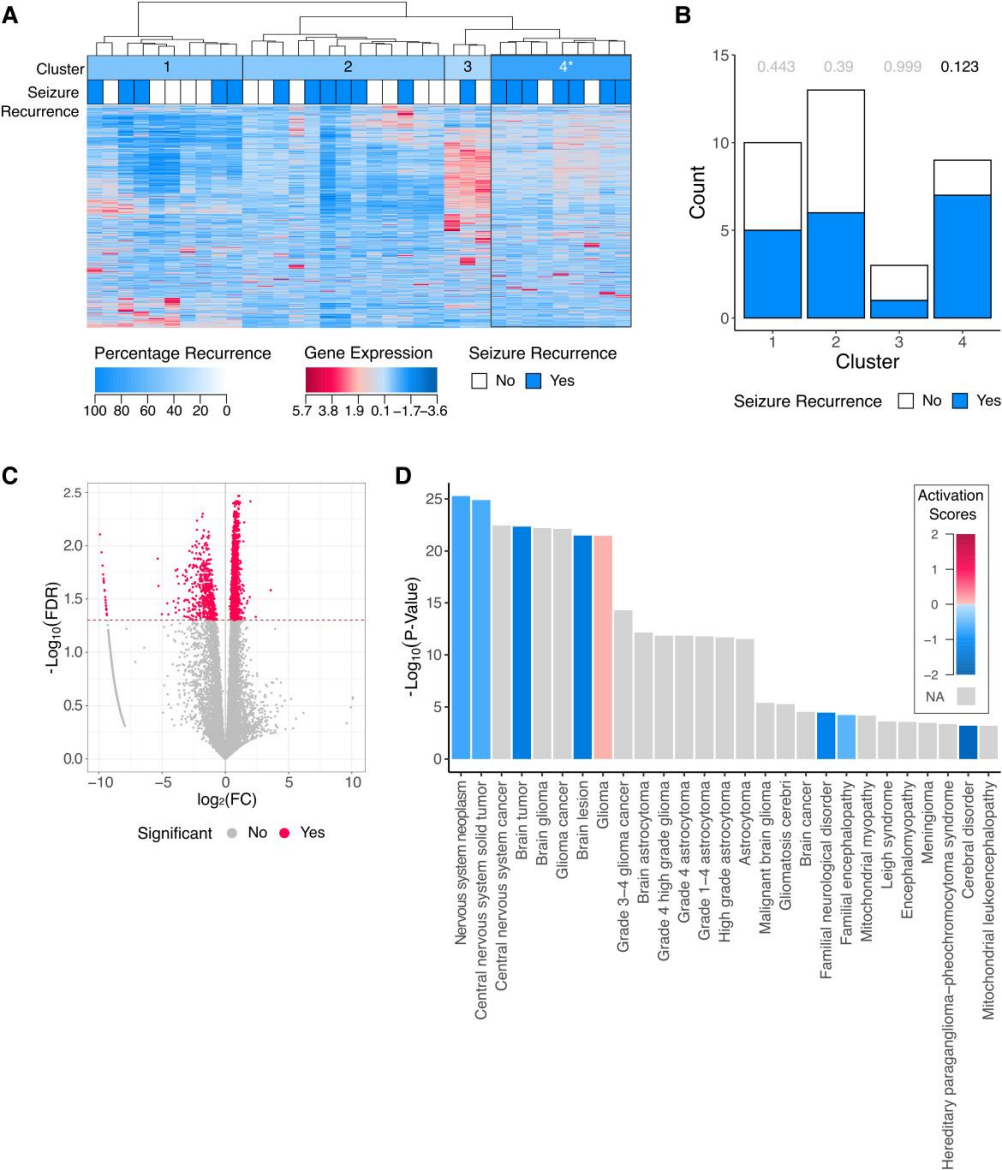
Identification of molecular subtype of epilepsy (B4), associated with seizure recurrence using RNA-Seq data. (**A**) Semi-supervised hierarchical clustering of mRNA transcript quantifications identifies cluster of individuals with a higher rate of seizure recurrence after surgical resection (cluster B4, multiple logistic regression, FDR *P* = 0.12). (**B**) The number of individuals in each cluster who experienced seizure freedom or seizure recurrence after surgical resection (multiple logistic regression, cluster B4 FDR *P* = 0.12). (**C**) Volcano plot depicting FC and FDR of 16 682 genes compared between cluster B4 and clusters B1–B3 (Student’s *t*-test, dotted line indicates FDR *P* = 0.05). (**D**) The neurological disease pathways (defined by IPA) that are enriched for the genes which are significantly associated with cluster B4, shaded by predicted pathway activation score (right-tailed Fisher’s exact test, *P* < 0.05).

Hierarchical clustering of mRNAs from blood samples using Canberra distance and Ward D similarity identified clusters most significantly associated with seizure outcomes, and four clusters were determined to be optimal (FDR < 0.20) (B1–4) (Fig. 3A; Supplementary Fig. 4A and B). No clusters associated with seizure recurrence were identified at more stringent FDR thresholds. Cluster B4 was associated with increased likelihood of seizure recurrence compared to clusters B1–B3 combined. In cluster B4, 78% (7/9) of individuals experienced seizure recurrence compared to 42% (11/26) of individuals in clusters B1 + B2 + B3 [78%, 95% CI (44–100%), FDR *P* = 0.12] (Fig. 3B; Supplementary Table 3). A total of 2486 of 16 683 mRNAs were significantly different between cluster B4 and individuals in other clusters (FDR *P* < 0.05) (Fig. 3B; Supplementary Table 5). These mRNAs were significantly enriched in 28 neurological disease pathways (*P* < 0.05) (Fig. 3D), and the top five most significantly enriched pathways were nervous system neoplasm, central nervous system solid tumour, central nervous system cancer, brain tumour, and brain glioma.

### mRNA and miRNA signatures between brain and blood in patient subtypes

**Figure 4:**
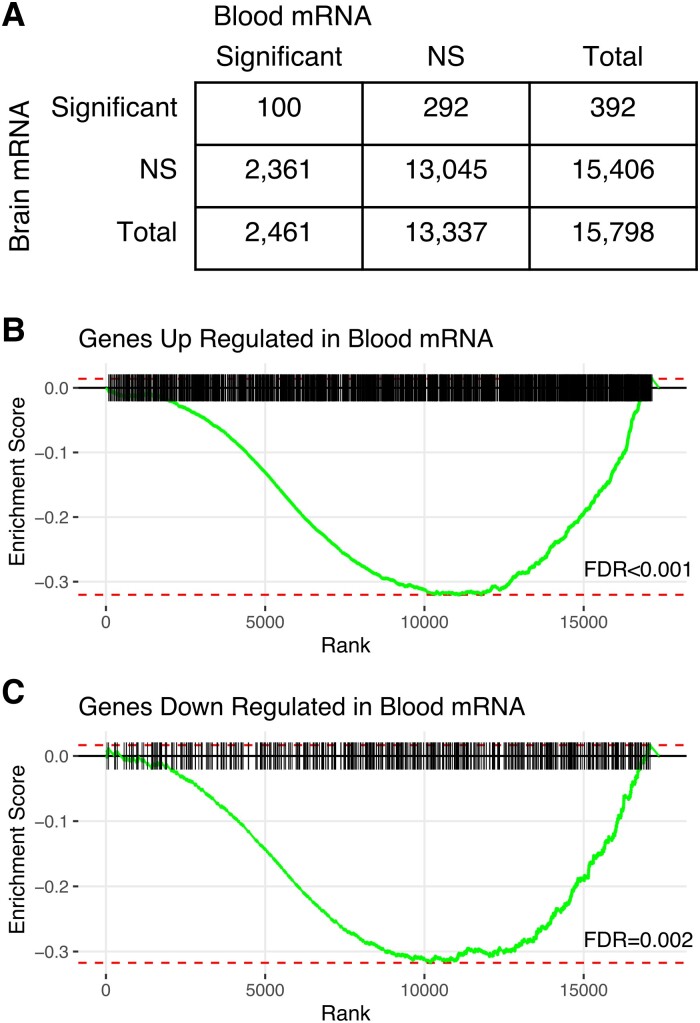
Gene expression in brain tissue of individuals in cluster B4. (**A**) Overlap of genes in blood significantly associated (Student’s *t*-test, FDR < 0.05) with cluster B4 and genes in brain tissue significantly associated with cluster B4 (Student’s *t*-test, FDR < 0.05). (**B**) Enrichment plot from gene expression analysis in which the genes upregulated in blood samples of cluster B4 are tested for enrichment among genes ranked by expression in brain tissue of cluster B4 (Gene Set Enrichment Analysis, Kolmogorov–Smirnov test, FDR *P* = 1.5 × 10^−9^). (**C**) Enrichment plot from gene expression analysis in which the genes downregulated in blood samples of cluster B4 are tested for enrichment among genes ranked by expression in brain tissue of cluster B4 (Gene Set Enrichment Analysis, Kolmogorov–Smirnov test, FDR *P* = 1.8 × 10^−3^).

Although clustering and subtype identification were performed on blood-based mRNA and miRNA, we examined the association of brain tissue–derived miRNA and mRNA transcripts with clusters A1 and B4. DE analysis of RNA-Seq performed on a subset of the TLE brain tissue samples (*n* = 30) collected from individuals who had both mRNA brain and tissue samples identified 392 genes that were significantly associated with cluster B4 membership when compared to the rest of the cohort (clusters B1 + B2 + B3) (*P* < 0.05) (Fig. 4A; Supplementary Table 6). Notably, 100 of these DE genes were also significantly different in the blood samples of cluster B4 (Fisher’s exact test, *P* = 3.0 × 10^−7^). The upregulated blood-derived genes associated with cluster B4 were found to be significantly enriched at the bottom of the tissue FC-ranked genes (FDR *P* = 1.5 × 10^−9^). Downregulated blood-derived genes associated with cluster B4 were also found to be significantly enriched at the bottom of the tissue FC-ranked genes (FDR *P* = 1.8 × 10^−3^). DE of miRNAs in temporal lobe brain tissue was assessed in 32 individuals of the miRNA cohort. There were no brain tissue miRNAs that were significantly associated with cluster A1 when compared to the rest of the cohort (clusters A2 + A3) (Supplementary Fig. 5A and Table 7). Similarly, blood-derived miRNAs associated with cluster A1 were not significantly enriched at the top or bottom of cluster A1 brain tissue FC-ranked miRNAs (Supplementary Fig. 5B).

**Figure 5:**
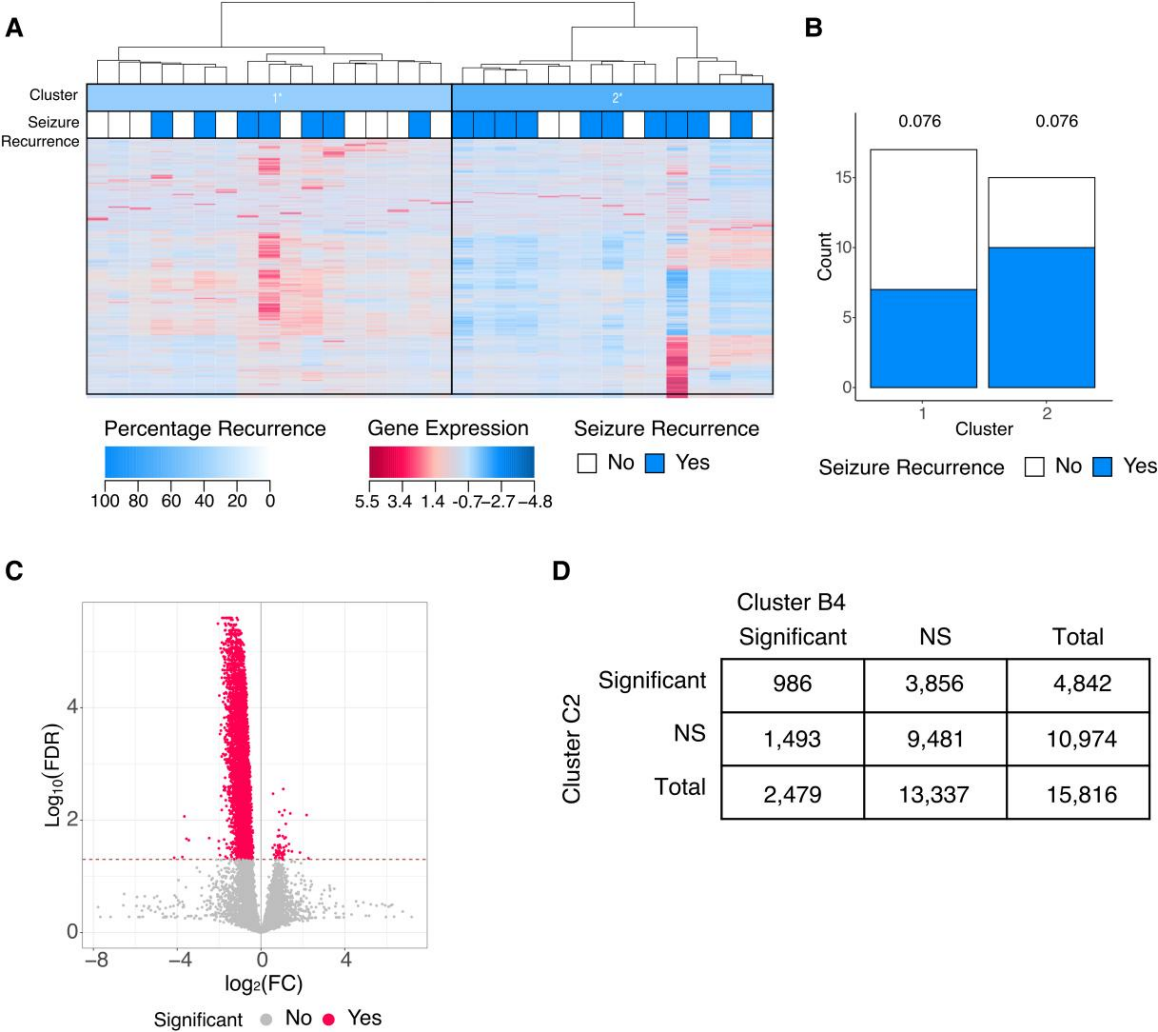
Identification of molecular subtype of epilepsy (C2), associated with seizure recurrence using RNA-Seq data. (**A**) Semi-supervised hierarchical clustering of brain tissue mRNA transcript quantifications identifies cluster of individuals with a higher rate of seizure recurrence after surgical resection (cluster C2, multiple logistic regression, FDR *P* = 0.08). (**B**) The number of individuals in each cluster who experienced seizure freedom or seizure recurrence after surgical resection (multiple logistic regression, FDR *P* = 0.08). (**C**) Volcano plot depicting FC and FDR of genes compared between cluster C2 and cluster C1 (Student’s *t*-test, dotted line indicates FDR *P* = 0.05). (**D**) Overlap of genes significantly associated with blood-derived cluster B4 and brain-derived cluster C2 (Fisher’s exact test, *P* < 2.2 × 10^−16^).

We further performed hierarchical clustering of brain tissue miRNA and brain tissue mRNA but did not identify any miRNA clusters associated with seizure outcomes (FDR > 0.20) (Supplementary Fig. 6). The clustering of mRNA from the brain tissue using Ward D similarity and Canberra distance metrics optimized at two clusters (Supplementary Fig. 6). Cluster C1 was associated with seizure freedom [41% seizure recurrence, 95% CI (18–65%), FDR *P* = 0.08], and C2 was associated with seizure recurrence [67%, 95% CI (47–87%), FDR *P* = 0.08] (Fig. 5A and B; Supplementary Table 3). There were 4824 mRNA transcripts that were significantly differentially expressed between clusters C1 and C2 (FDR *P* < 0.05) (Fig. 5C; Supplementary Table 8). Of these, 968 transcripts were also significantly altered in the blood-based mRNA B4 cluster, resulting in a statistically significant overlap (*P* < 2.2 × 10^−16^) (Fig. 5D).

### Patient overlap between blood and brain-derived mRNA and miRNA subtypes

**Figure 6:**
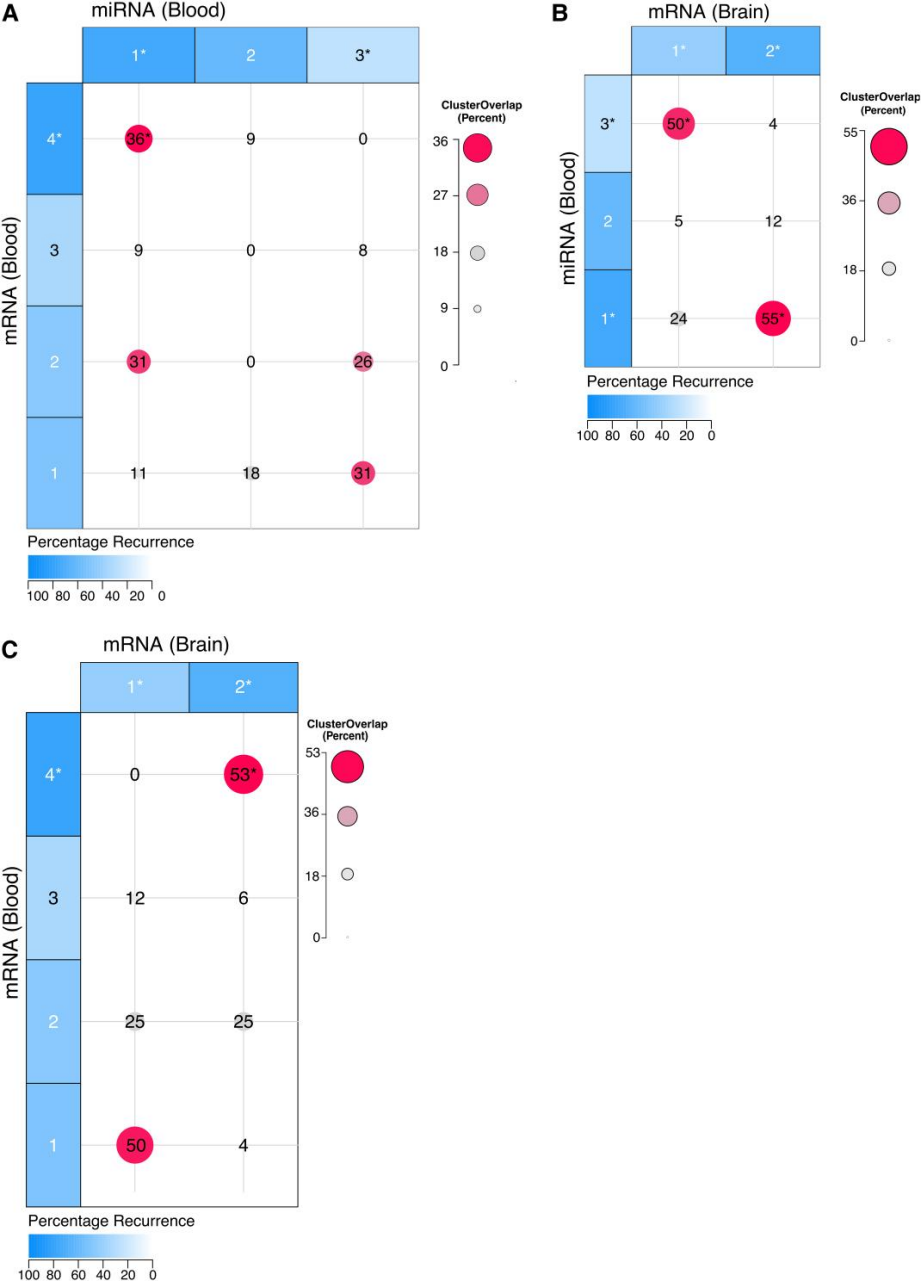
Overlap of patients between each pair of clusters from blood miRNA, blood mRNA and brain mRNA. Clusters are shaded by the percentage of individuals who experienced seizure recurrence after surgical resection, and asterisks denotes that (**A**) cluster A1 and cluster B4 are significantly overlapping (Fisher’s exact test, *P* = 0.04), (**B**) clusters A3 and C1 are significantly overlapping (Fisher’s exact test, *P* = 0.006), clusters A1 and C2 are significantly overlapping (Fisher’s exact test, *P* = 0.03), and (**C**) clusters B4 and C2 are significantly overlapping (Fisher’s exact test *P* = 0.001). The size and colour of the circle and label indicate the percentage of overlap between each pair of clusters.

There were 30 individuals who were included in each of the blood miRNA (*n* = 40), blood mRNA (*n* = 35), and brain mRNA (*n* = 32) cohorts. We assessed the overlap of all clusters that were significantly associated with seizure recurrence (A1, B4, and C2) and seizure freedom (A3 and C1). The premise is that clusters containing a common set of individuals based on separate analyses of miRNA and mRNA signatures that are similarly over- or under-represented for an outcome is more likely to represent a common molecular aetiology, representing an endophenotype. Significant overlap of individuals with miRNA and mRNA signatures that are associated with seizure recurrence or freedom could indicate a common disease aetiology. Of the 35 individuals with both blood mRNA and blood miRNA data, miRNA cluster A1 (*n* = 21) and mRNA cluster B4 (*n* = 9) had significant overlap; eight individuals were present in both clusters (36% overlap, *P* = 0.04) (Fig. 6A). Of the 32 individuals with both brain tissue mRNA and blood miRNA data, brain-derived mRNA cluster C1 (*n* = 17) and blood-derived miRNA cluster A3 (*n* = 10), which were both significantly enriched for seizure freedom, had significant overlap of nine individuals (50% overlap, *P* = 0.006) (Fig. 6B). Brain-derived mRNA cluster C2 (*n* = 15) and blood-derived miRNA cluster A1 (*n* = 19) were associated with seizure recurrence and shared 12 individuals (55% overlap, *P* = 0.03) (Fig. 6B). Of the 30 individuals with both blood and brain tissue mRNA data, blood-derived cluster B4 (*n* = 8) and brain-derived cluster C2 (*n* = 15) which were associated with seizure recurrence were significantly overlapping (53% overlap, *P* = 0.001) (Fig. 6C). Of the 30 individuals who had brain tissue mRNA, blood mRNA, and blood miRNA, seven individuals were present in cluster A1, cluster B4, and cluster C2, and six individuals were present in two of the three clusters’ association with seizure recurrence (Supplementary Fig. 7).

### Blood mRNA and miRNA expression suggests a coordinated mechanism

**Figure 7:**
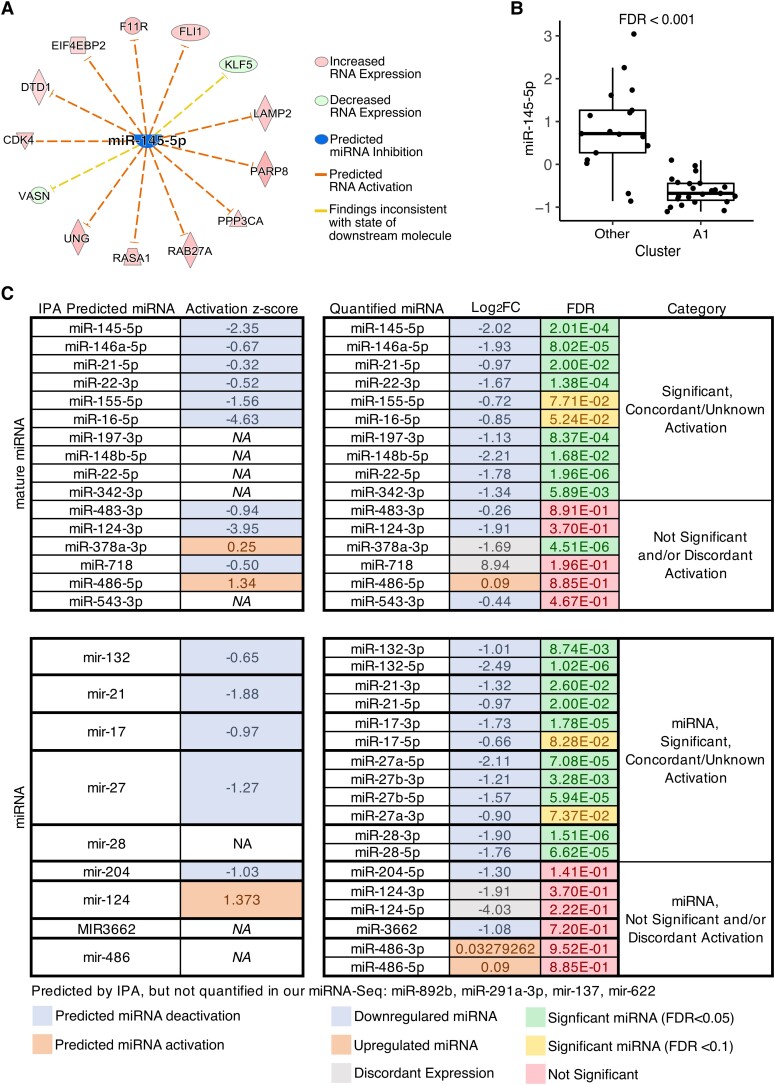
Intersection of cluster A1 and B4 miRNA and mRNA networks. (**A**) Based on the gene expression patterns in mRNA cluster B4, miR-145-5p is predicted to be deactivated in cluster B4 (Fisher’s exact test, *P* = 0.039, activation *Z*-score = −3.95). (**B**) miR-145-5p is downregulated in miRNA cluster A1 (Student’s *t*-test, FDR = 2 × 10^−4^). (**C**) Columns 1 and 2: list of miRNAs predicted to be activated or deactivated based on gene expression patterns observed in mRNA cluster B4 (Fisher’s exact test, *P* = <0.05). Columns 3–5: list of miRNA expression levels (log_2_FC) and significance (FDR, Student’s *t*-test) in miRNA cluster A1. Expression levels that are concordant with predicted activation levels are coloured by increased expression and decreased expression, and miRNAs with expression levels that are discordant with the predicted miRNA activation are shaded accordingly. Significance values from the Student's *t*-test are shaded to indicate level of significance; FDR *P* < 0.05, FDR *P* < 0.1, FDR *P* > 0.1.

mRNA expression patterns observed in cluster B4 were predicted to interact with several miRNAs. For example, 11/13 mRNAs known to be suppressed by miR-145-5p were upregulated in mRNA cluster B4, and therefore, miRNA-145-5p was predicted to be deactivated (Fig. 7A). Interestingly, we observed that in miRNA cluster A1, which significantly overlapped with individuals in mRNA B4, miRNA-145-5p was significantly downregulated (log_2_FC = −2.02, FDR *P* = 2 × 10^−4^) (Fig. 7B). In total, 10 mature miRNAs and five precursor miRNA hairpins that were predicted to be upstream modulators of the mRNA cluster B4 associated genes were found to be significantly downregulated in miRNA cluster A1 (FDR *P* < 0.1) (Fig. 7C).

## Discussion

A reported 40–80% of individuals with TLE who undergo surgical resection will experience seizure recurrence.^3–5^ Using clinical variables such as imaging and electrophysiology data to identify candidates for whom surgical resection will provide seizure freedom has improved the surgical outcome for many patients.^8–10^ However, molecular signatures associated with seizure recurrence have the potential to improve existing predictive models and advance our mechanistic understanding of surgical outcomes. Identifying these molecular signatures through easily accessible peripheral blood samples collected pre-operatively will transform clinical decision-making.

In this study, we used a novel clustering approach to identify molecular subtypes of epilepsy associated with seizure recurrence using two sources of transcriptomic data, mRNA transcripts and miRNAs, collected from the peripheral blood and brain of individuals who underwent resective epilepsy surgery. The three subtypes at higher risk for post-operative seizure recurrence shared a significant overlap of individuals, suggesting that there may be a common signature comprised of both mRNAs and miRNAs in the blood and brain tissue that delineates those who will experience seizure recurrence from those who experience seizure freedom post-resection. Pathway analysis identified overlapping mRNA/miRNA networks between the two subtypes, further supporting the existence of a common molecular mechanism driving seizure recurrence.

Despite being identified in the peripheral blood, the miRNAs identified in cluster A1 are known regulators of many neurological disease pathways, including epilepsy, suggesting molecular mechanisms by which these miRNAs may contribute to seizure recurrence. Almost all miRNAs associated with cluster A1 were downregulated, suggesting that these miRNAs in the blood may be anticonvulsant, although additional mechanistic work will be needed to better characterize the relationship between these miRNAs and seizures. Similarly, many of the mRNA transcripts that define cluster B4 were also significantly altered in the brain, highlighting a potential source of mechanistically informed transcripts in the blood. The collection of mRNAs and miRNAs associated with these subtypes is comprised of novel and known targets involved in seizure recurrence.

Of the 455 miRNAs that were differentially expressed between cluster A1 and clusters A2–A3, many were over-represented in epilepsy-related pathways such as chronic epilepsy, kainic acid–induced epileptic seizures, and epilepsy or neurodevelopmental disorder, among others. Among the 10 miRNAs most significantly different between clusters A1 and A2–A3, miR-132-5p (FDR *P* < 0.001, log_2_FC −2.49) and miR-22-5p (FDR *P* < 0.001, log_2_FC −1.78) were both downregulated. miR-132-5p has been observed to be upregulated in the hippocampus of rats displaying both epilepsy onset in infancy and adult acute, latent, and chronic epilepsy. miR-132-5p has also been studied in the context of other neurological disorders, such as Alzheimer’s disease, Parkinson’s disease and amyotrophic lateral sclerosis (ALS).^32–34^ miR-22 is enriched in the brain; deletion of miR-22 has been shown to reduce inflammation after seizures but also exacerbates seizures, suggesting a role for miR-22 in the regulation of neuroinflammation after seizures to reduce epilepogenesis.^35^ miR-22 also plays a role in other neurological disorders including Alzheimer’s disease, Huntington’s disease, cerebral ischaemia, ALS, multiple sclerosis, panic disorders, schizophrenia and traumatic brain injury.^36^

Over 2000 genes were differentially expressed between cluster B4 and cluster B1. Among these genes, those involved in neurological disease were over-represented, although the majority of the significant pathways were related to glioma, astrocytoma and other brain cancers. However, *PIGB*, which is implicated in hereditary epileptic seizures, is among the top 10 genes driving the separation of cluster B4 (FDR *P* = 0.003, log_2_(FC) = 1.02).^37,38^ Notably, *PIGB* was also significantly altered in cluster C2 (FDR *P* = 0.048, log_2_(FC) = −0.783). One hundred genes that were differentially expressed in blood samples of cluster B4 were also differentially expressed in the brain tissue samples of individuals in cluster B4. Among these, *KLHL15* (tissue: log_2_FC = −2.1, FDR *P* = 0.009; blood: log_2_FC = −1.5, FDR *P* = 0.03) has been observed to be partially deleted in a case study of an individual with a severe intellectual disability, epilepsy and anomalies of cortical development.^39^ Additionally, the mitochondrial gene *Vdac2* (tissue: log_2_FC = −0.92, FDR *P* = 0.03; blood: log_2_FC = −1.33, FDR *P* = 0.04) is associated with refractory epilepsy in pharmacoresistant rats.^40^

Over 4000 genes were differentially expressed between cluster C2 and cluster C1. The 10 most significant genes included *AP2B1* [FDR *P* = 2.5 × 10^−6^, log_2_(FC) = −1.6], also significant in cluster B4 [FDR *P* = 0.021, log_2_(FC) = .02], a gene involved in endocytosis and autophagy that plays a role in dendritogenesis^41^ and has been implicated in Alzheimer’s disease.^42^*DNAJB9*, a gene involved in cellular response, was significantly lower in cluster C2 [FDR *P* = 1.7 × 10^−9^, log_2_(FC) = −1.5]. *Dnajb9* in the midbrain of mice is associated with longevity,^43^ and expression is increased in the spinal cord fluid of individuals with ALS.^44^ A translocation affecting *ITFG1* has been reported in an individual with epilepsy and severe neurodevelopmental delay. This gene was differentially expressed in both cluster C2 [FDR *P* = 2.52 × 10^−9^, log_2_(FC) = −1.15] and cluster B4 [FDR *P* = 0.009, log_2_(FC) = 0.79]. *PPP1R7* is significantly differentially expressed in cluster C2 [FDR *P* = 2.53 × 10^−6^, log_2_(FC) = −1.66] and cluster B4 [FDR *P* = 0.004, log_2_(FC) = 0.73] and is part of 2q37 microdeletion syndrome, a disease in which 35% of individuals experience seizures.^45^

IPA generated a list of miRNAs predicted to be significantly altered upstream of the DE genes observed in cluster B4. There was a large overlap in these predicted miRNAs and the DE miRNAs in cluster A1. Among the overlapping miRNAs, miR-145-5p has shown potential as a biomarker of epilepsy in serum.^46^ miR-146a-5p has been implicated in rat models of epilepsy and childhood epileptic encephalopathies.^47,48^ miR146a-5p has also been observed to be up- or downregulated in the CNS or blood of individuals with Alzheimer’s disease, ALS, multiple sclerosis and dementia.^49^ miR-21-5p has been shown to be protective of hippocampal neurons in a rat epilepsy model by inhibiting *Stat3* expression and is also commonly differentially expressed in multiple sclerosis, Alzheimer’s disease, ALS, age-related macular degeneration, ataxia, Parkinson’s disease and myotonic dystrophy.^47,49^ miR155-5p is a regulator of inflammation in the brain and other tissues. In TLE patients, miR-155 is correlated with seizure frequency, and individuals with high expression of hippocampal miR-155 have a shorter duration of post-resection seizure freedom.^50^ Additional work in a rat model of epilepsy showed that the absence of miR-155-5p correlated with an increase in sestrin-3 expression and was associated with decreased seizures and apoptosis in the hippocampus.^50^ Other researchers have shown that delivery of a miR-155-5p antagonist decreases seizure-induced pentetrazol administration in a rat model of epilepsy.^51^ Additionally, this miRNA plays a role in Alzheimer’s disease, ALS, age-related macular degeneration, multiple sclerosis, Parkinson’s disease, dementia and glaucoma.^49^ Finally, miR-204 has been observed to be downregulated in human tissue mesial TLE and hippocampal sclerosis.^52^

All the blood and brain tissue samples collected for this study were from individuals who were diagnosed with drug-resistant epilepsy. While some of these mRNAs and miRNAs are part of molecular pathways that represent the hallmarks for neurological disease, we found that these target transcripts and miRNAs are differentially expressed in a subtype of epileptic patients who experience post-operative seizure recurrence. Therefore, although the mechanism by which some of these miRNAs and mRNAs contribute to seizure recurrence may be shared by other neurological disorders, our study suggests that these biomarkers may be used collectively to identify groups of patients who will be less responsive to surgical resection.

The findings of this study were limited by sample size, and future work is planned to reproduce the results in a larger cohort after additional samples are collected. While the clusters were significantly enriched for individuals with seizure recurrence, it is difficult to interpret the heterogeneity that remains within clusters, and this will require additional research. A larger cohort will allow us to develop a predictive model using both the mRNA transcripts and miRNAs identified here. We will also investigate the role of these miRNAs and mRNA transcripts to better understand mechanisms driving seizure recurrence on a molecular level.

In summary, this pilot study demonstrates the potential of integrating multi-omics data for identifying molecular subtypes of epilepsy that associate with seizure recurrence using peripheral blood samples and has highlighted several mRNA transcripts and miRNAs that may contribute to post-resection seizure recurrence. This approach shows substantial promise to identify molecular factors involved in seizure outcome and may lead to prediction models to improve the identification of surgical resection candidates.

## Supplementary material

Supplementary material is available at *Brain Communications* online.

## Supplementary Material

fcad251_Supplementary_Data

## Data Availability

The data that support the findings of this study are not publicly available because of IRB-based restricted access. Sharing of data with a qualified researcher may be permissible with IRB approval and a data use agreement. Further information about the data sets is available from the corresponding author upon reasonable request.
