# Supplementary material for: Molecular subtypes of epilepsy associated with post-surgical seizure recurrence
Source: Brain Commun. 2023 Sep 30;5(5):fcad251. doi: 10.1093/braincomms/fcad251 (PMC10597540; doi:10.1093/braincomms/fcad251)
Supplement: fcad251_Supplementary_Data [file fcad251_supplementary_data.zip › Supplementary_figures_and_supplementary_table_legends.docx]

**Supplementary Material**

**Molecular Subtypes of Epilepsy associated with Post-Surgical Seizure-Recurrence**

**B**

**A**

**
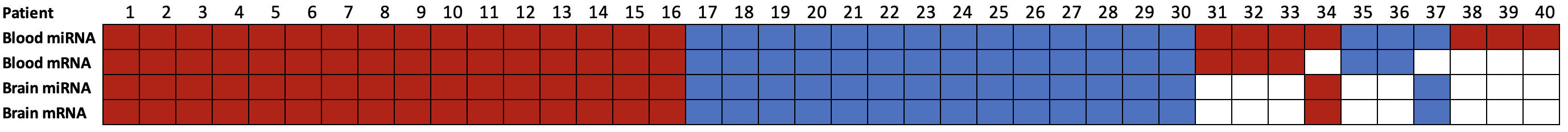
**

**
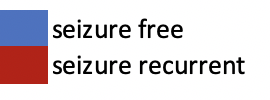
**

**Supplementary Figure 1. Cohort Characteristics** (A) Distribution of the number of days between blood sample collection and surgical resection. (B) Patients in each molecular cohort. Red indicates patient experienced seizure recurrence and pre-operative sample was collected and processed. Green indicates that patient did not experience seizure recurrence and sample was collected and processed. White indicates that samples were not collected. 30 individuals were in all four cohorts.

**Supplementary Figure 2. Example of method to selection of optimal clusters.**  The dendogram was partitioned and each cluster compared to the rest of the cohort and tested for an association with seizure recurrence using multiple logistic regression. At the first split (2 clusters), a significant cluster was identified (Cluster 2, logistic regression, *P* = .02). Next, the dendogram was split again and each cluster was tested for an association with seizure recurrence. Of the three clusters, Cluster 3 had a lower nominal *P* value (logistic regression, *P* = 0.0008) than any of the clusters from the previous partition, therefore the dendogram was split again into 4 clusters. Each of the four clusters were tested for an association with seizure recurrence, but none of the resulting nominal *P* values were smaller than Cluster 3 of the previous partition. Therefore, the optimal number of clusters was determined to be three.


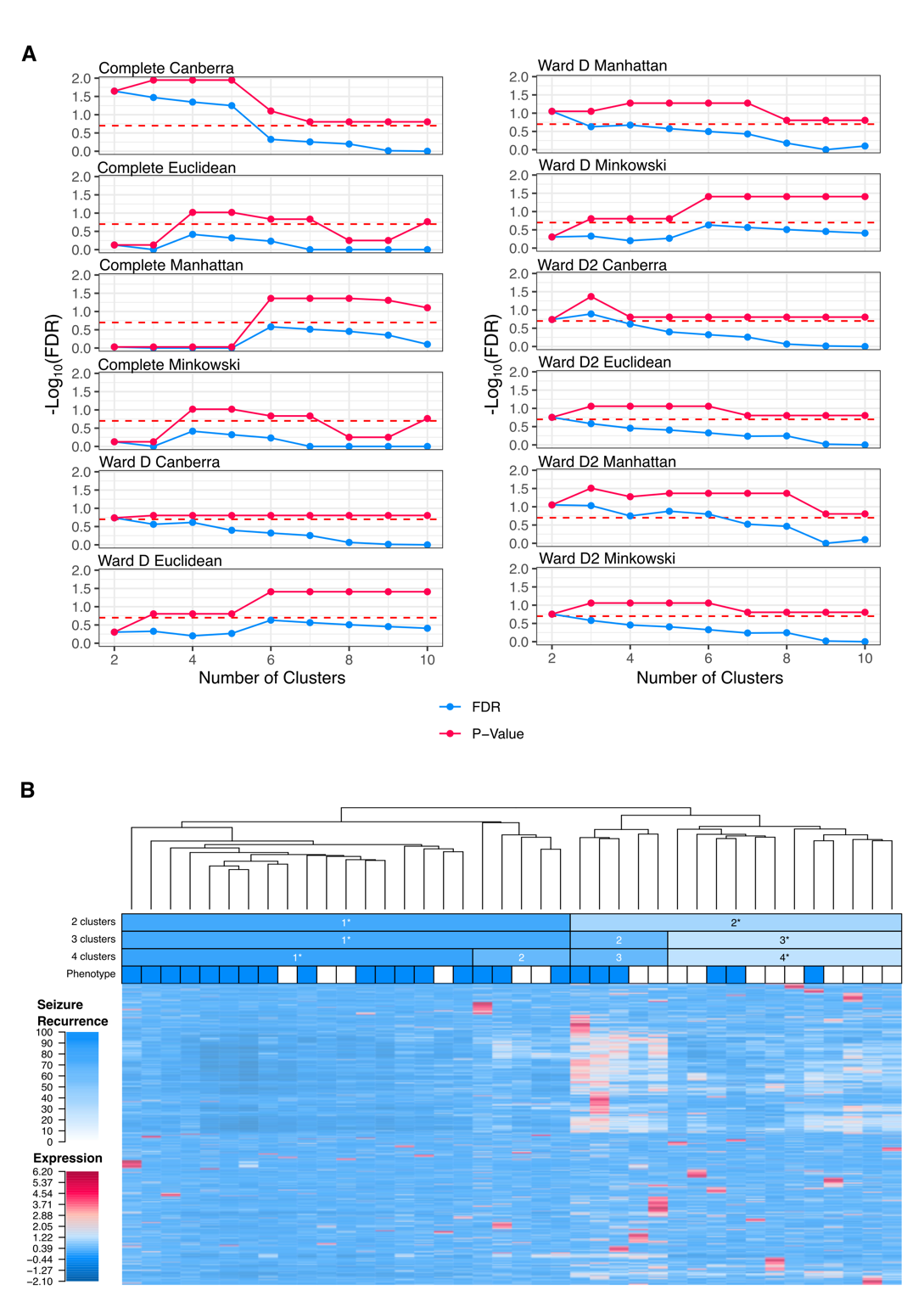


**Supplementary Figure 3. Optimization of hierarchical clustering to identify genetic subtypes of epilepsy using miRNA expression.** A) For each similarity and distance metric, the significance (logistic regression, P-Value and FDR) of the cluster with the strongest association with seizure recurrence is displayed across 2-10 clusters. B) The heatmap depicts the clustering of miRNA expression (similarity metric=complete, distance metric=Canberra). The dendogram displays the similarity between individuals, while the annotation shows the clusters; the proportion of seizure recurrence within each cluster (color), and the significance of the association with seizure recurrence (asterisks, logistic regression, FDR *P*<0.2) across 1-3 splits. The optimal number of clusters was determined to be three.


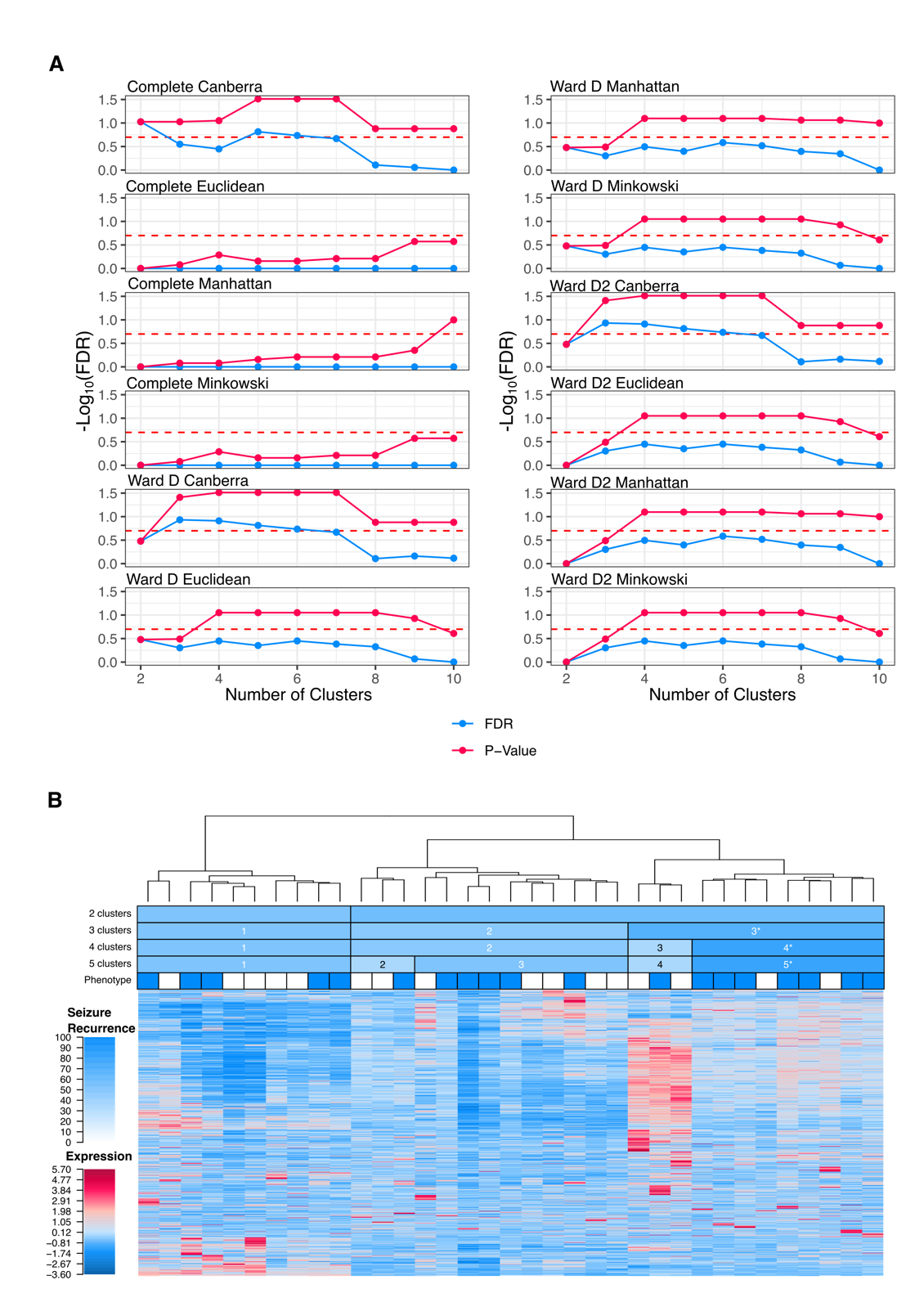


**Supplementary Figure 4. Optimization of hierarchical clustering to identify genetic subtypes of epilepsy using mRNA expression.** A) For each similarity and distance metric, the significance (logistic regression, P-Value and FDR) of the cluster with the strongest association with seizure recurrence is displayed across 2-10 clusters. B) The heatmap depicts the clustering of mRNA expression (similarity metric=Ward D2, distance metric=Canberra). The dendogram displays the similarity between individuals, while the annotation shows the clusters; the proportion of seizure recurrence within each cluster (color), and the significance of the association with seizure recurrence (asterisks, logistic regression, FDR *P*<0.2) across 1-4 splits. The optimal number of clusters was determined to be four.


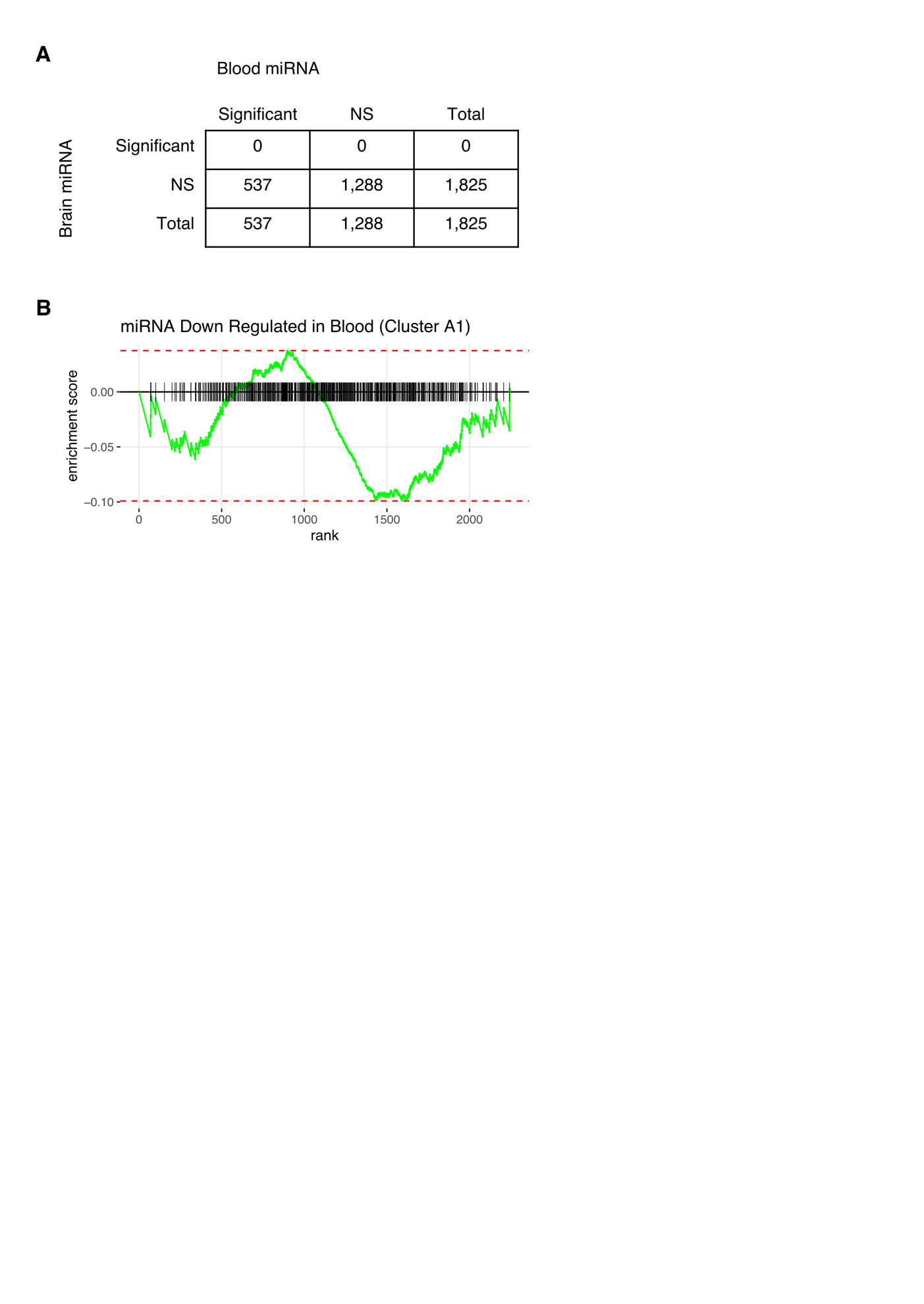


**Supplementary Figure 5. miRNA expression in brain tissue of individuals in Cluster A1.** A) Overlap of miRNA in blood significantly associated (Student’s T-test, FDR < 0.05) with cluster A1 and miRNA in brain tissue significantly associated with cluster A1 (Student’s T-test, FDR<0.05). B) Enrichment plot from gene expression analysis in which the miRNAs downregulated in blood samples of cluster A1 are tested for enrichment among miRNA ranked by expression in brain tissue of cluster A1 (GSEA, Kolmogorov-Smirnov test).

**A**

**B**

**
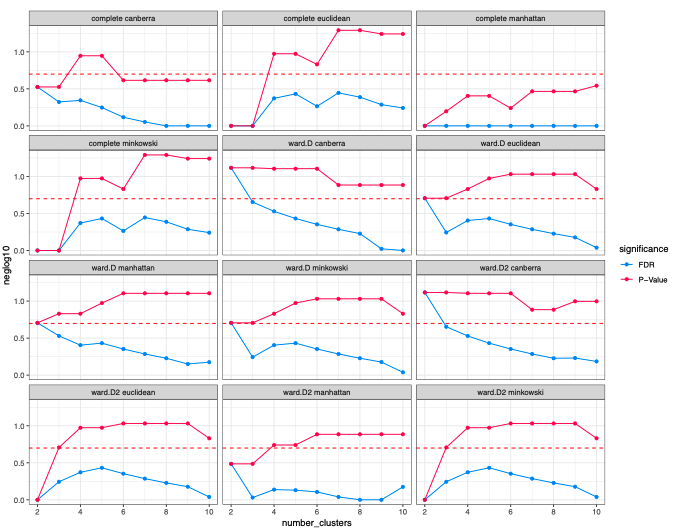
**

**Supplementary Figure 6. Optimization of hierarchical clustering to identify genetic subtypes of epilepsy using miRNA and mRNA expression in brain tissue samples.**  For each similarity and distance metric, the significance (P-Value and FDR) of the cluster with the strongest association with seizure recurrence is displayed across 2-10 clusters for A) brain tissue miRNA and B) brain tissue mRNA. The subtyping algorithm optimized at two clusters for brain tissue mRNA (logistic regression, FDR *P*=0.076)(similarity metric=Ward D, distance metric=Canberra).

**
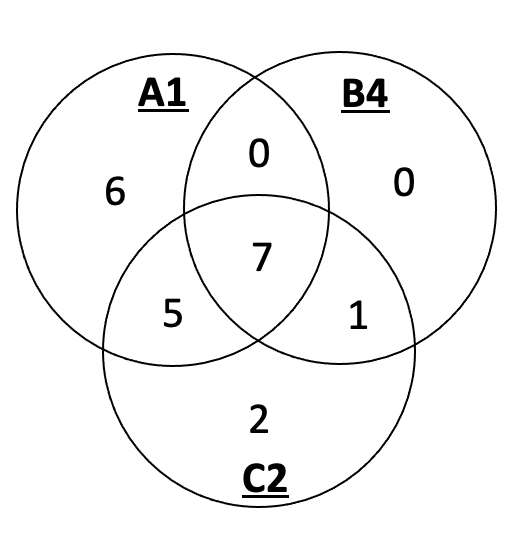
**

**Supplementary Figure 7.** 30 patients were in each of the three blood miRNA, blood mRNA, and brain mRNA cohorts. The Venn Diagram indicates the number of patients in cluster A1, B4, and C2 associated with seizure recurrence, and the overlap of these individuals.

**Supplementary Table 1:** Patient ASM prescription records at time of blood draw and time of surgery.

**Supplementary Table 2:** The number of patients on each ASM at time of blood draw and time of surgery.

**Supplementary Table 3:** Bootstrapped proportions of seizure recurrence, standard deviations within each significant cluster compared to the rest of the cohort.

**Supplementary Table 4:** Significance and magnitude of change for each miRNA from blood samples tested for differences in expression between cluster A1 and all other clusters.

**Supplementary Table 5:** Significance and magnitude of change for each mRNA from blood samples tested for differences in expression between cluster B4 and all other clusters.

**Supplementary Table 6:** Significance and magnitude of change for each mRNA from brain tissue tested for differences in expression between cluster B4 and all other clusters.

**Supplementary Table 7:** Significance and magnitude of change for each miRNA from brain tissue tested for differences in expression between cluster A1 and all other clusters.
